# Common occurrence of Sharpey’s fibres in amphibian phalanges

**DOI:** 10.1007/s00435-018-0400-4

**Published:** 2018-02-15

**Authors:** Krzysztof Kolenda, Anna Najbar, Beata Rozenblut-Kościsty, Ewa Serwa, Tomasz Skawiński

**Affiliations:** Department of Evolutionary Biology and Conservation of Vertebrates, Faculty of Biological Sciences, University of Wrocław, Sienkiewicza 21, 50-335 Wrocław, Poland

**Keywords:** Bone histology, Enthesis, Muscle, Phalanx, Tendon

## Abstract

Sharpey’s fibres are known mainly as providing anchorage between tooth and the periodontal ligament but they occur also in other types of bones. In the postcranial skeleton these fibres are usually present at the muscle or tendon attachment sites. They were reported in all major groups of extant vertebrates, as well as in putative lissamphibian ancestors—temnospondyls and lepospondyls. However, it was recently stated that their presence was very rarely described in extant amphibians. In limbs, they were reported predominantly from proximal bones. They have not yet been reported from phalanges, which are the most commonly sectioned amphibian bones. Here, we describe phalangeal histology of nine species representing most major clades of lissamphibians. These results show that Sharpey’s fibres occur commonly in lissamphibian phalanges. In shaft, they are radially oriented and occur in the periosteal bone, at sites of tendon attachment. They can also occur in the metaphysis and contact the cartilage. This may provide a basis for foot muscle reconstructions in fossil amphibians.

## Introduction

Sharpey’s fibres (SF) are poorly mineralised fibres of the connective tissue, composed mostly of several types of collagen, elastin or tenascin (e.g. Francillon-Vieillot et al. 1990; Aaron 2012). They are known primarily as providing attachment between tooth and a periodontal ligament (e.g. Ho et al. 2007). However, they occur also in other body parts, where they most commonly attach muscles, ligaments or tendons to collagen fibres present in the periosteal bone (Francillon-Vieillot et al. 1990; Aaron 2012) but can also be present in osteoderms, in which they probably serve as anchorage to the skin (e.g. Witzmann and Soler-Gijón 2010). Some authors even restrict the definition of SF only to collagen fibres inserting the bone (Simmons et al. 1993; after Hall 2015) but it seems that this definition is less commonly used than the broader one (Aaron 2012; Hall 2015). Although the presence of the periosteal SF is less well documented than those involved in tooth anchorage, they have been described in most of the major groups of extant tetrapods, such as mammals (Singh et al. 1974; Aaron 2012; Warshaw et al. 2017), birds (Genbrugge et al. 2012; Petermann and Sander 2013) and reptiles (Castanet et al. 1988; Hutchinson 2002; Suzuki et al. 2002, 2003). Recently, Clemente-Carvalho et al. (2009) described SF from the skull of brachycephalid frogs and stated that this is only the second reference to SF in amphibians, besides the report of their occurrence in the femur of the American bullfrog *Lithobates catesbeianus* (Felisbino and Carvalho 2000). However, these fibres have also been described in the osteoderms of some frogs (Ruibal and Shoemaker 1984; Quinzio and Fabrezi 2012), the stylopodial bones of the conrauid frog *Conraua goliath* and the cryptobranchiid salamander *Andrias davidianus* (Canoville et al. 2018), the skull of an extinct cryptobranchiid *Eoscapherpeton asiaticum* (Skutschas and Boitsova 2017) and in the vertebrae of an extant caecilian *Ichthyophis kohtaoensis* (Castanet et al. 2003). Interestingly, SF have been more commonly described in fossil stem-amphibians than in living ones, including both dermal and endochondral bones of both temnospondyls and lepospondyls (e.g. Witzmann 2009; Konietzko-Meier and Sander 2013; Danto et al. 2016)—two groups which may be ancestral to lissamphibians, a clade including all living amphibians (Marjanović and Laurin 2013).

Studying phalanges is less invasive than using more proximal bones, which requires sacrificing the life of an individual, and thus is preferred particularly in endangered species, such as most lissamphibians. Thus, phalanges are the bones most commonly used in the histological age determination of amphibians (i.e. skeletochronology; Sinsch 2015). Despite the fact that phalanges were used in skeletochronological studies on wide range of extant amphibians, to our knowledge the presence of SF has not yet been reported in those bones. The aim of our study was to find out whether SF occur commonly or rarely in lissamphibian phalanges. This could also provide an experimental basis for inferring muscle attachment sites in fossil amphibians. Numerous muscles attach to the amphibian foot, including several attaching to the fourth digit, responsible for its flexion and extension. Most of them have tendinous attachment sites (entheses), where SF are usually particularly abundant.

For this purpose we investigated bone histology of nine species which belong to several major clades of extant amphibians (Lissamphibia) and represent different modes of life and locomotion (Table 1)—factors which may affect bone histology (e.g. Laurin et al. 2004; Canoville and Laurin 2009).

**Table 1:** Lifestyle and mode of locomotion of the studied species (lifestyle according to Laurin et al. 2004)

Scientific name	Vernacular name	Lifestyle	Locomotion
*Salamandra salamandra*	Fire salamander	Terrestrial	Walking
*Lissotriton vulgaris*	Smooth newt	Amphibious	Walking, swimming
*Bombina bombina*	European fire-bellied toad	Aquatic	Walking, swimming
*Pelobates fuscus*	Common spadefoot toad	Terrestrial	Walking, burrowing
*Bufo bufo*	European common toad	Terrestrial	Walking
*Bufotes viridis*	European green toad	Terrestrial	Walking
*Hyla arborea*	European tree frog	Terrestrial	Walking, arboreal
*Rana temporaria*	European common frog	Amphibious	Jumping
*Pelophylax ridibundus*	Marsh frog	Amphibious	Jumping, swimming

## Materials and methods

### Taxon and specimen sampling

**Fig. 1:**
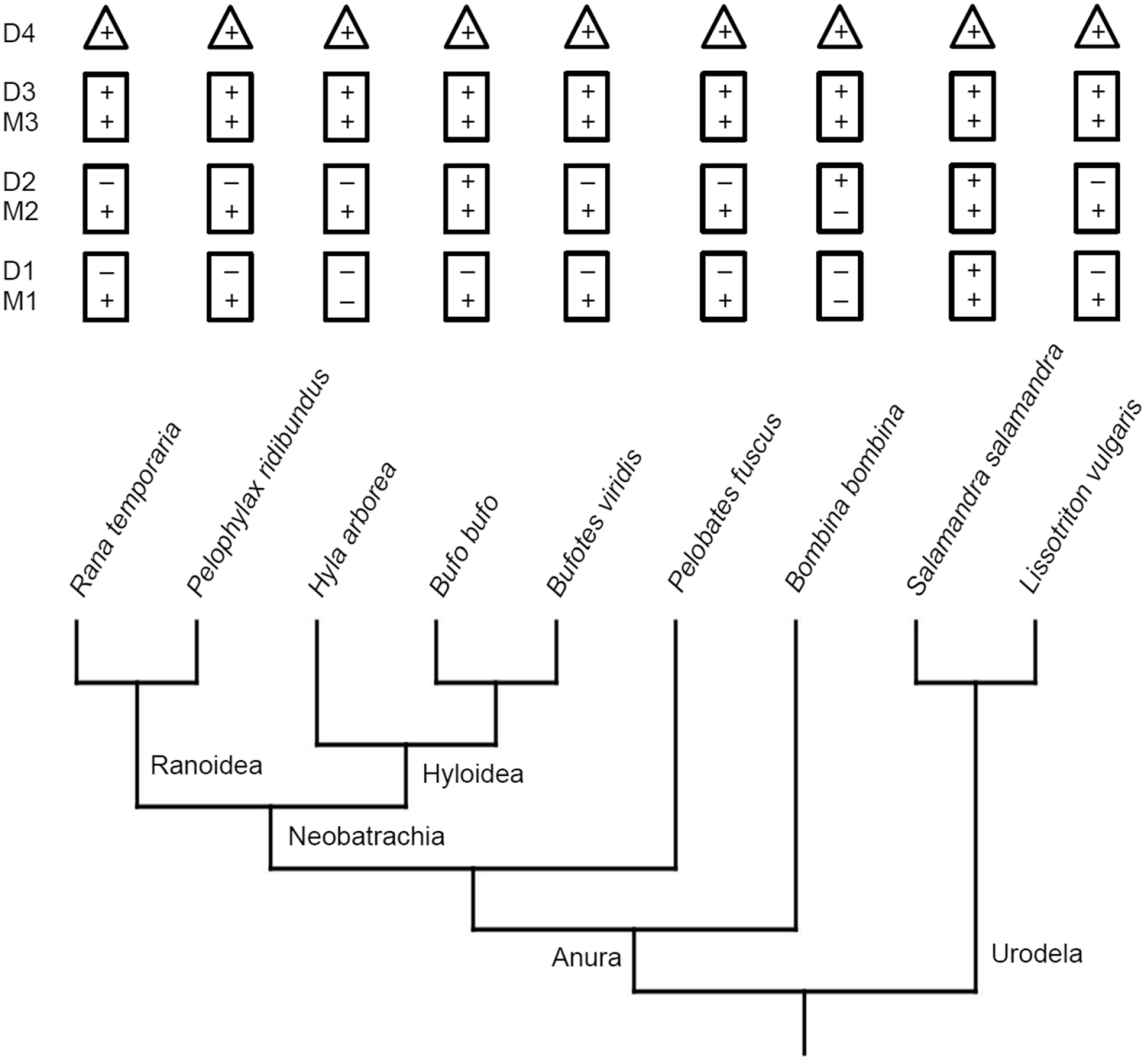
Simplified dendrogram showing relationships of species used in this study (after Frost et al. 2006; Pyron and Wiens 2011). It shows also the occurrence of Sharpey’s fibres in the phalanges of the fourth toe of the hindlimb. *M* metaphysis, *D* diaphysis (*M1* metaphysis of the first phalanx, *D2* diaphysis of the second phalanx and so on). “+” observed, “−” not observed

The sample consisted of adult individuals of *Bombina bombina, Bufo bufo, Bufotes viridis, Hyla arborea, Pelobates fuscus, Pelophylax ridibundus, Rana temporaria, Salamandra salamandra* and *Lissotriton vulgaris* (see Fig. 1 for their phylogenetic relationships). Each of these species was represented by three specimens (with the exception of *H. arborea*, of which two specimens were available). All of them come from the collections of the Department of Evolutionary Biology and Conservation of Vertebrates, University of Wrocław and the Institute of Zoology, Poznań University of Life Sciences. No animal was killed for the purpose of this study.

### Specimen preparation and analysis

The fourth digit of the right hind limb was taken (in contrary to other digits, it has four phalanges). It was manually cleaned of associated soft tissues. The phalanges were decalcified in a 1:1 mixture of 10% formic acid and 4% formalin for 1–4 h, depending on the size of the bone. To remove traces of decalcifying agent, bones were washed in four changes of distilled water, 15 min each, and stored in 70% ethanol. We embedded the bones in a tissue freezing medium (Leica, Biosystems Nussloch GmbH, Germany) and sectioned into 10 µm thick sections on a freezing microtome (Leica CM 1850 UV), and stained with 0.05% cresyl violet, following Rozenblut and Ogielska (2005). In all cases phalanges were sectioned from proximal to distal epiphysis.

We tested also several other methods of sectioning and staining, utilised commonly in histological studies, using third phalanx of *B. bufo* as an example. All phalanges were dehydrated, cleared in xylene, then embedded into paraffin, sectioned using Leica RM2255 microtome into 7 µm thick sections and mounted on slides. Several different methods of staining were tested: (1) Mallory’s solution, in which cartilage and bone stain red and collagen fibres—deep blue; (2) Mallory’s trichrome, in which cartilage and bone matrix stain blue and collagen fibres—dark blue (3) and Delafield’s haematoxylin and eosin, in which collagen fibres stain pink due to the acid aniline eosin. However, these methods performed more poorly than the one utilising freezing microtome and staining with cresyl violet. Additionally, unstained sections were observed under polarised light and by dark-field microscopy. The sections were analysed under a light microscope (Carl Zeiss Axioskop 20) at different magnifications (ranging from 20× to 40×).

We used Burton (2004) as a model for amphibian pes musculature.

## Results

### Bone histology

**Fig. 2:**
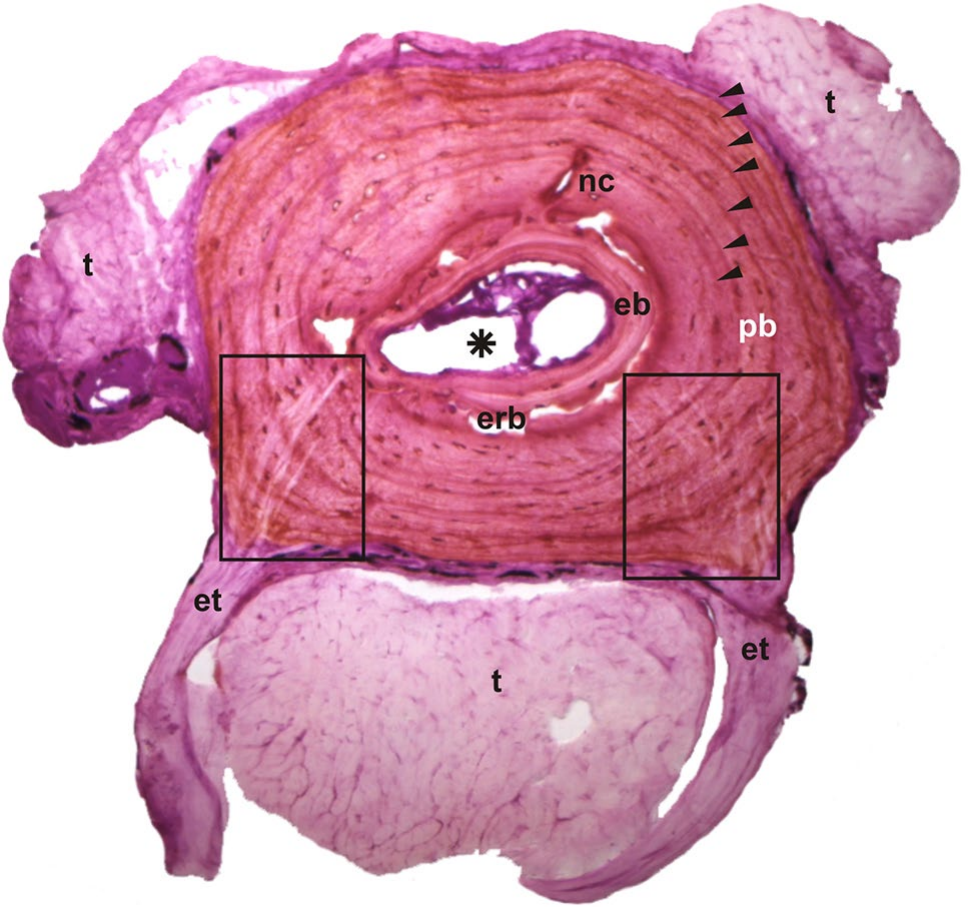
Transverse section of the third phalanx (diaphysis) of *Bufotes viridis*. Black squares—location of Sharpey’s fibres, black arrows—lines of arrested growth, asterisk—medullary cavity. *t* Tendon, *et* enthesis, *eb* endosteal bone, *nc* nutrient canal, *pb* periosteal bone, *erb* erosion bay

The compacta was the thickest in the middle part of the diaphysis and the medullary cavity expanded toward the epiphyses. The bone tissue was parallel-fibred in most cases but in some thin-sections of salamander bones we observed a lamellar bone. Lines of arrested growth (LAGs) which reflect cessation of bone growth during hibernations were well visible and their number confirmed that all studied individuals were adult (actively growing animals may differ in the histological structure of the bone; Castanet et al. 2003; Canoville and Laurin 2009). The bones were rather poorly vascularised (e.g. *B. viridis* in Fig. 2) or avascular. In the periosteal bone of *S. salamandra, L. vulgaris* and *P. fuscus* there was a single, large nutrient canal, in *B. bombina* we observed two nutrient canals on the opposite parts of the periosteal bone, while neobatrachians (ranids, bufonids and *Hyla*) tended to show larger number of nutrient canals. The degree of resorption of the periosteal bone and apposition of endosteal bone determined the size of the medullary cavity in the mid-diaphysis. The largest cavities were observed in *B. bufo, B. viridis* and *H. arborea*, while other species had much narrower cavities. In bufonids, the margin of the medullary cavity was irregularly resorbed, creating erosion bays. In *H. arborea* there was a balance between bone resorption and apposition, resulting in nearly oval and smooth borders of the cavity. In *B. bufo, B. viridis* and *H. arborea* the endosteal bone formed only a thin ring around the medullary cavity. The other species had better developed endosteal bone that filled large parts of the resorbed cavity. The smallest degree of resorption was observed in *S. salamandra, L. vulgaris* and *B. bombina*.

### Sharpey’s fibres

**Fig. 3:**
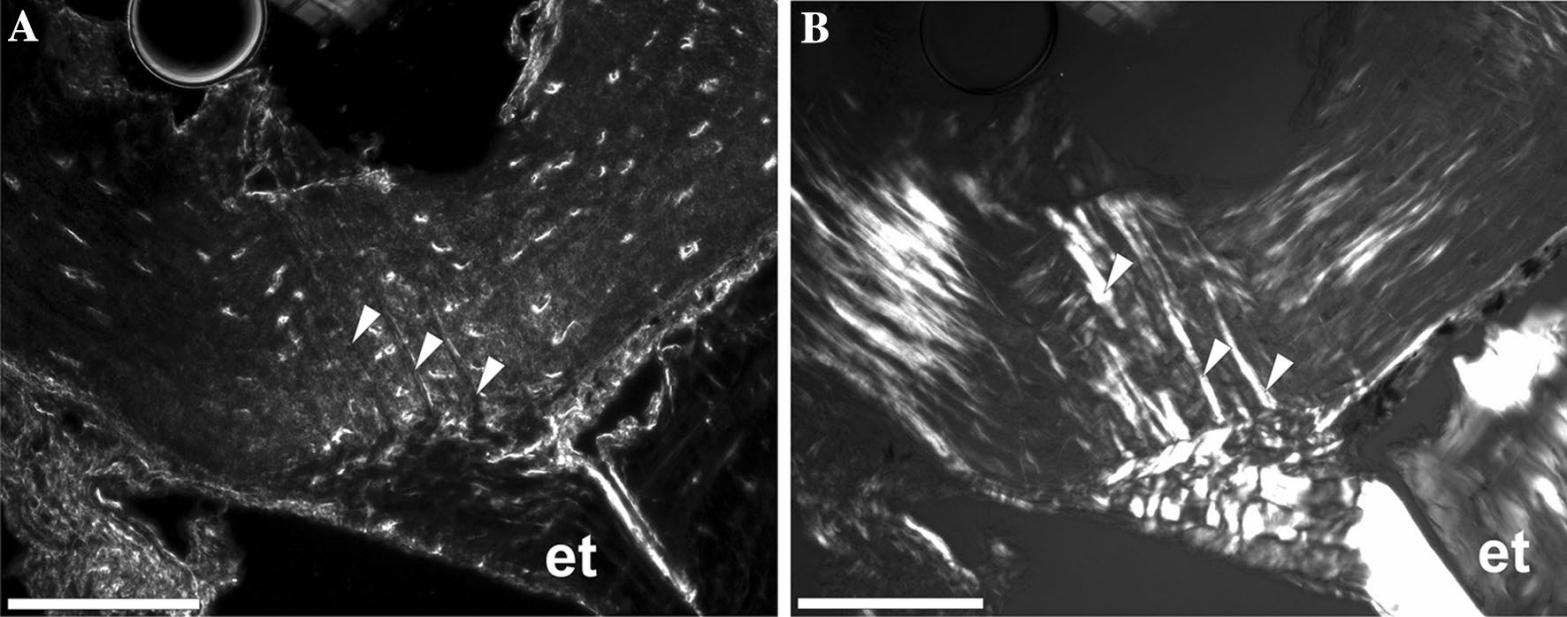
Diaphysis of the third palanx of *B. bufo* seen under dark-field (**a**) and polarised light (**b**). White arrows—Sharpey’s fibres, *et* enthesis for *M. lumbricalis brevis*. Scale bar equals 100 µm

**Fig. 4:**
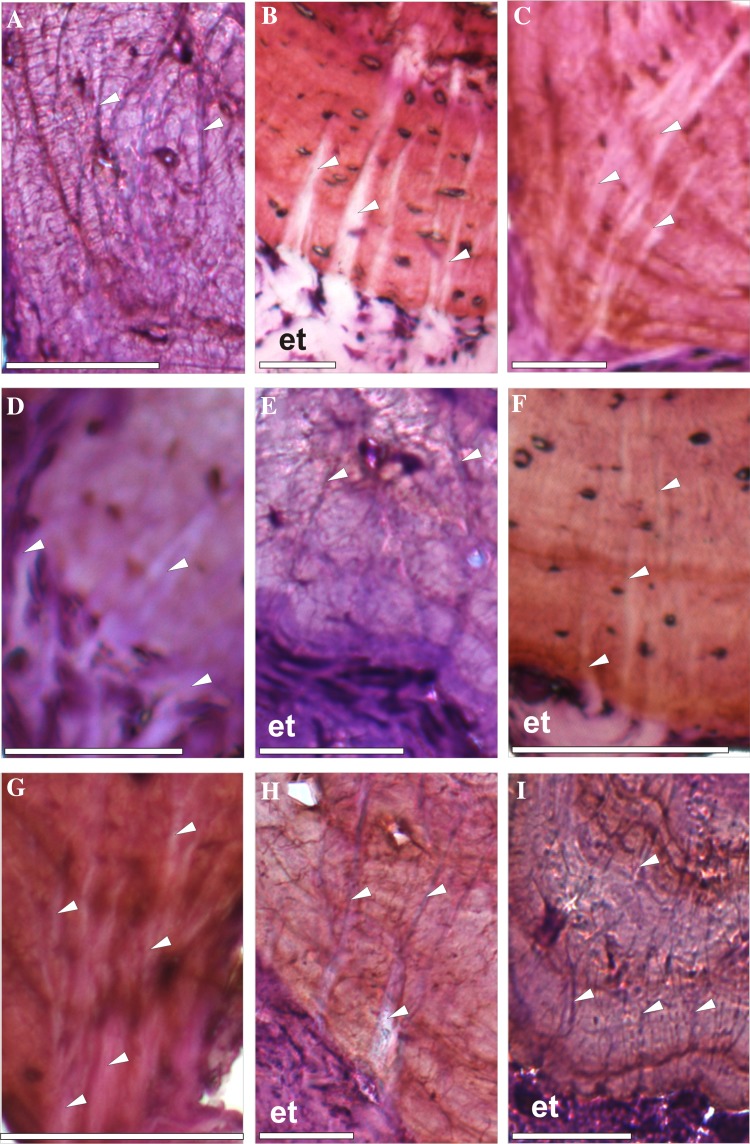
Sharpey’s fibres in transverse sections of the third phalanx (diaphysis) of: **a** *Bombina bombina*, **b** *Bufo bufo*, **c** *Bufotes viridis*, **d** *Hyla arborea*, **e** *Pelobates fuscus*, **f** *Pelophylax ridibundus*, **g** *Rana temporaria*, **h** *Salamandra salamandra*, **i** *Lissotriton vulgaris*. White arrows—Sharpey’s fibres, *t* tendon. Scale bar equals 100 µm. Dorsal side upwards

**Fig. 5:**
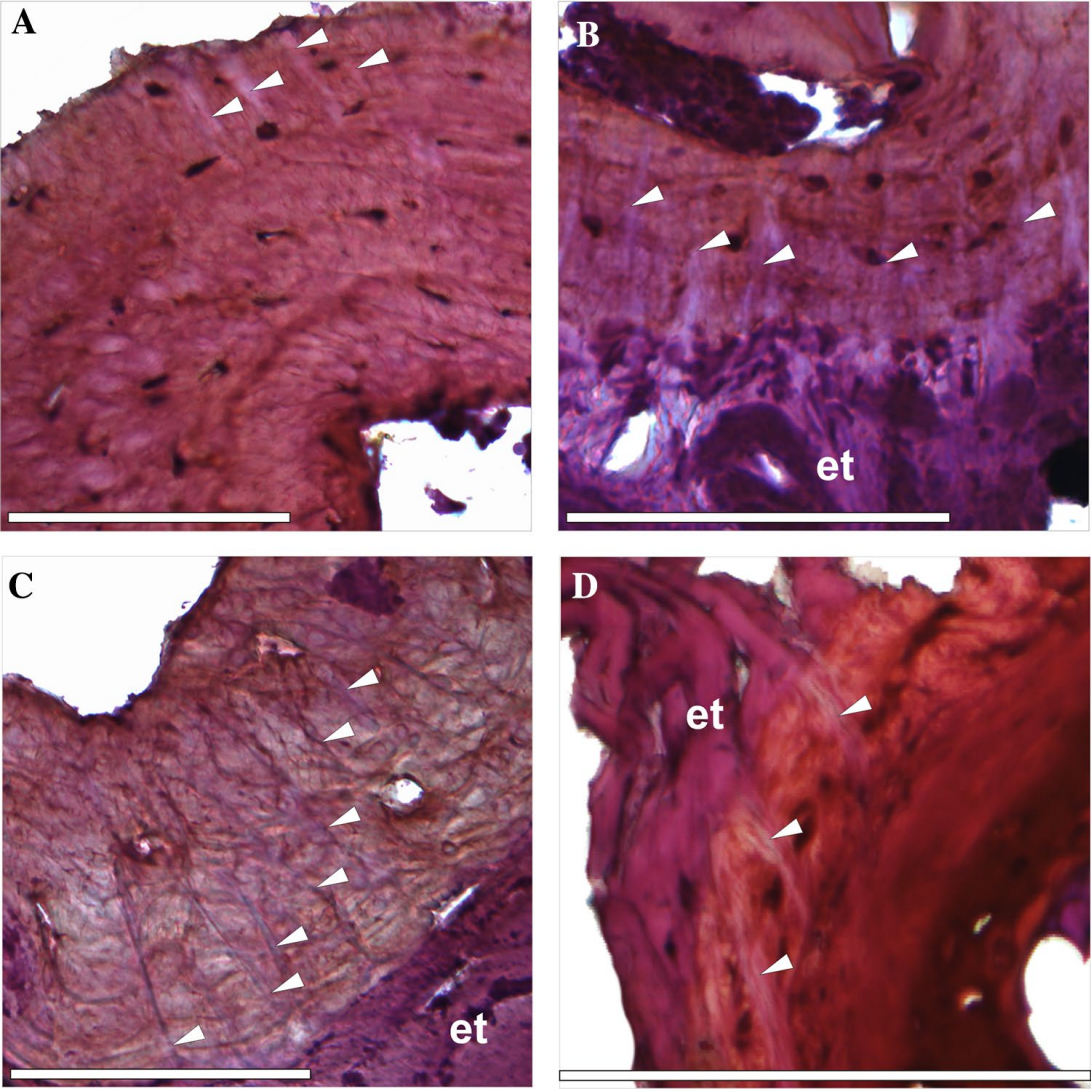
Sharpey’s fibres in different phalanges: **a** second phalanx (diaphysis) of *B. bufo* with SF located on the left side, **b** fourth phalanx (diaphysis) of *H. arborea* with SF located both on the ventral and the dorsal side, **c** first phalanx (diaphysis) of *S. salamandra* with SF located on the ventral side, **d** metaphysis of the third phalanx of *R. temporaria* with SF located on the lateral side. White arrows—Sharpey’s fibres, *et* enthesis. Scale bar equals 100 µm. Dorsal side upwards

Distinct bundles of fibres occurred in phalanges of all sampled species, most commonly in the II and III phalanges. However, they were not observed in all phalanges of every individual (Fig. 1). In the diaphysis, the fibres were radially oriented, running perpendicularly or slightly obliquely to the bone. They were most numerous in the bony ridges, where tendons attach, and where the periosteal SF occur most commonly (morphologically, they are most similar to type 1 SF in Konietzko-Meier and Sander 2013). They were well visible under dark-field and strongly birefringent under polarised light in *B. bufo* (Fig. 3) but the birefringence was more poorly characterised in *S. salamandra*. In the proximal phalanx of anurans, they were particularly well developed on the ventral side of the metaphysis, where medial *M. lumbricalis brevis* had tendinous attachment. In urodeles, SF were present also on the dorsal side of the bone, at sites where *M. extensoris brevis superficialis* and *M. extensor brevis profundus* attached indirectly to the bone. In the second phalanx they also occurred on the ventral side of the metaphysis, where *M. lumbricalis longus*, lateral *M. lumbricalis brevis*, and *M. interphalangealis proximalis* had their tendinous attachment sites. In urodeles, SF also occurred on the dorsal surface, where lateral *M. extensoris brevis superficialis* attached to the phalanx through the tendon. In the third phalanx, we did not observe SF on the dorsal side in anurans but they were present in the urodeles. On the ventral side, the fibres were present in all examined species, in both shaft and metaphysis, where *M. interphalangealis distalis* and medial *M. lumbricalis brevis* have entheses. In the distal phalanx, SF are present on ventral and dorsal surfaces in both anurans and salamanders. To this bone, *M. extensor brevis distalis* and *tendo superficialis* are attached. SF may contact the medullary cavity or the endosteal bone (if present) but we did not observe them penetrating the endosteal bone (Fig. 4). SF may also be present in the metaphysis, where they can contact the hyaline cartilage (Fig. 5). They penetrated the bone beneath the epiphysis and between metaphysis and lateral articular surface, and are present on both dorsal and ventral sides. The best developed SF were present in two toad species, while in *Bombina* they were only faintly visible. In the places where SF occurred, growth marks were difficult to observe.

## Discussion

The intraspecific differences in bone tissue type observed in some species are not surprising. The histological bone structure may be affected by many factors, both internal and external, such as individual age, sex and environmental or geographic aspects (e.g. Alcobendas and Castanet 2000; Castanet et al. 2003; Kaczmarski et al. 2016).

Presence of SF is well documented in many types of bones (both cranial and postcranial, dermal and endochondral) of Palaeozoic and Mesozoic amphibians, including both temnospondyls and lepospondyls (e.g. Witzmann 2009; Mukherjee et al. 2010; Sanchez et al. 2010; Konietzko-Meier and Klein 2013; Konietzko-Meier and Sander 2013; Canoville and Chinsamy 2015; Danto et al. 2016; Gruntmejer et al. 2016). At least one of these two groups gave rise to lissamphibians (see review in Marjanović and Laurin 2013). However, histological studies on fossil lissamphibians are rare and SF have not yet been reported in extinct caudates (bone histology of fossil salientians is even more poorly studied, so no comparison can be made), except in the frontal of the Late Cretaceous cryptobranchiid *Eoscapherpeton* (de Buffrénil et al. 2015; Skutschas and Stein 2015; Skutschas and Boitsova 2017). The discovery that SF are common in extant amphibians strongly suggests that their presence in extinct amphibians of similar mode of life is expected (at least in phalanges). Recently, Petermann and Sander (2013) presented an experimental basis for inferring muscle attachment sites based on histological sections of amniote femora. Our study provides an extension to Lissamphibia and may serve as a basis for future studies investigating different parts of the skeleton.

Clemente-Carvalho et al. (2009) claimed that before their publication SF were mentioned only in Felisbino and Carvalho (2000), but these fibres are clearly visible in figures provided by some other authors (e.g. Guarino et al. 2011; Sinsch 2015). However, they were unlabelled, even in those in which other histological structures have been described (e.g. Sinsch 2015).

In two species of toads and in *Salamandra*, which are among the most terrestrial of lissamphibians, SF were developed more prominently (i.e. the fibres were thicker and covered greater area of the section) than in species spending more time in water, such as the fire-bellied toads (compare Fig. 4b, c, g with a). Thus, one could suspect that this may be related to greater forces acting on the limbs during terrestrial locomotion. However, SF are very well developed in femora and humeri of the aquatic Chinese salamander *Andrias davidianus* (Canoville et al. 2018), as well as in the Triassic temnospondyl *Metoposaurus krasiejowensis*, which is interpreted as almost exclusively aquatic (nonetheless, this amphibian was probably able to burrow—this requires strong muscles, which would be consistent with the presence of well developed SF; Konietzko-Meier and Sander 2013). On the other hand, we did not observe well developed SF in *P. fuscus*, despite partially burrowing lifestyle of this amphibian. However, in closely related *P. varaldii* these fibres can readily be observed in at least some specimens (Guarino et al. 2011). Also, it should be noted that the presence of SF may be dependent on a number of physiological stimuli, at least in mammals. These include influence of hormones (such as estrogen), degree of physical activity, ageing or pathologies such as osteoporosis or osteoarthritis (Aaron 2012). Explaining the reasons of these differences in amphibians requires further studies.

The fact that SF may obscure some of the growth marks, such as LAGs, potentially hinders skeletochronological studies, especially when only incomplete bones are available (e.g. Castanet et al. 1988). Thus, for skeletochronological analyses it may be advisable to use—if possible—the proximal rather than ultimate or penultimate phalanges, as these phalanges have relatively weakly developed SF.
